# Golden Syrian Hamster Models for Cancer Research

**DOI:** 10.3390/cells11152395

**Published:** 2022-08-03

**Authors:** Zhongde Wang, Robert T. Cormier

**Affiliations:** 1 Department of Animal, Dairy, and Veterinary Sciences, Utah State University, Logan, UT 84322, USA; 2 Department of Biomedical Sciences, University of Minnesota Medical School, Duluth, MN 55812, USA

**Keywords:** golden Syrian hamster, cancer, *KCNQ1*, *TP53*, *IL2RG*

## Abstract

The golden Syrian hamster (*Mesocricetus auratus*) has long been a valuable rodent model of human diseases, especially infectious and metabolic diseases. Hamsters have also been valuable models of several chemically induced cancers such as the DMBA-induced oral cheek pouch cancer model. Recently, with the application of CRISPR/Cas9 genetic engineering technology, hamsters can now be gene targeted as readily as mouse models. This review describes the phenotypes of three gene-targeted knockout (KO) hamster cancer models, *TP53*, *KCNQ1*, and *IL2RG*. Notably, these hamster models demonstrate cancer phenotypes not observed in mouse KOs. In some cases, the cancers that arise in the KO hamster are similar to cancers that arise in humans, in contrast with KO mice that do not develop the cancers. An example is the development of aggressive acute myelogenous leukemia (AML) in *TP53* KO hamsters. The review also presents a discussion of the relative strengths and weaknesses of mouse cancer models and hamster cancer models and argues that there are no perfect rodent models of cancer and that the genetically engineered hamster cancer models can complement mouse models and expand the suite of animal cancer models available for the development of new cancer therapies.

## 1. History of Hamsters as Animal Models in Cancer Research

The golden Syrian hamster (*Mesocricetus auratus*) has been demonstrated to be especially effective in modeling human disorders such as diet-induced early atherosclerosis, inflammatory myopathies, emerging viral infectious diseases, *Clostridium difficile* infection, pancreatitis, diet-induced obesity, insulin resistance, and lipid metabolism [1,2,3,4,5,6,7,8]. Notably, for several decades hamsters have also been successfully used to model virally and chemically induced cancers [9,10,11,12,13,14,15,16,17,18,19,20,21]. An excellent example is oral cancers induced via topical administration of carcinogenic chemicals such as 7,12-dimethylbenz[a]anthracene (DMBA). Head and neck cancer is the sixth most common cancer worldwide with oral cancers comprising roughly half of all head and neck cancers, and despite advances in therapy morbidity and mortality for oral cancers remain high. For several decades the hamster model of sequential oral carcinogenesis following topical administration of DMBA to the hamster cheek pouch has been shown to be a superior animal model for recapitulation of human oral cancers, from premalignant to malignant states. The hamster oral cheek model is especially informative for oral cancers that develop from the use of smokeless tobacco products (STPs) that are chewed or dipped in the human cheek pouch. It is noted that there are an estimated 300 million STP users worldwide including 3% of US adults and 6% of US high school students [15]. For the treatment of the growing number of oral cancers, the hamster oral cheek model has also been effective in the development of new therapies such as boron neutron capture therapy (BNCT) [11,15,17,18,19,20]. Another example of a long-used effective chemically induced cancer in the hamster is the *N*-Nitrosobis(2-oxopropyl)amine (BOP)-treated model of pancreatic cancer [12,16]. Pancreatic cancer is an extremely lethal disease with close to 450,000 deaths each year worldwide and a poor 5-year survival rate of 10%. The hamster BOP model closely resembles the stages in human pancreatic cancer as it causes pancreatic intraepithelial neoplasia (PanIN), a premalignant stage of pancreatic cancer that progresses to severe pancreatic ductal adenocarcinoma as observed in humans, including harboring similar genetic mutations such as in *K-ras*, *CDKN2A*, and *SMAD4* [16]. Finally, another example of the long-standing use of the hamster in cancer research is its employment as an animal model of oncolytic adenoviruses and to evaluate new antiviral drugs [13].

However, until recently a significant challenge for the use of hamsters in modeling cancer and other human diseases, compared, for example, with mouse models, was the inability to generate genetically engineered hamsters. The first steps to overcome this limitation came with the full sequencing and annotation of the hamster genome and transcriptome [14,21]. Now, this barrier has been surmounted by our group, resulting in gene KOs and knockins in the hamster by employing CRISPR/Cas9-mediated gene targeting, piggyBac-mediated transgenesis, and pronuclear injection [22,23] (see the section below). In this review, we will also describe the phenotypes of the first three genetically engineered hamster cancer models, Kos in the tumor suppressors *TP53* and *KCNQ1*, and a KO in the *IL2RG* gene, whose first application was to provide a non-murine model to study X-linked severe combined immunodeficiency (XSCID) and the infectious diseases associated with IL2RG deficiency. Most recently, we have employed the *IL2RG* KO hamsters to create patient-derived xenograft (PDX) cancer models, in particular for metastatic pancreatic cancer.

## 2. Is There a Need for More Genetically Engineered Rodent Models of Cancer?

Given the long-standing use of mouse models of cancer, in particular genetically engineered mouse models (GEMM) that employ new complex technologies, it is a legitimate question to ask whether there is a need for new animal models, especially new rodent models of cancer. In this section, we will review how rodent models can help answer fundamental questions in human cancer biology and then compare the strengths and weaknesses of mouse models and hamster models. Our key argument is that there is no perfect rodent model for specific cancers and that by expanding the suite of cancer models in different species these models can complement each other and enhance their overall value as models of human cancer. We start by asking how rodent models can provide insights into human cancers.

What are the important issues and questions that animal/rodent models can provide insights into human cancers? Certainly, preclinical animal models are essential for the development of safe, effective therapies for human cancers. All therapeutic drugs that eventually enter the clinic are first tested in animal models for safety and efficacy. Notably, genetically engineered animal models have been extremely valuable in evaluating emerging precision cancer therapies. An example was the development of immune checkpoint inhibitors (ICIs) that were first tested in several types of mouse models (KO, immunodeficient, humanized, syngeneic) that recapitulate human immune responses to ICIs in vivo, including both efficacy [24,25,26] and the risk of adverse events [27]. At the cancer cell and molecular level, rodent models have been invaluable in understanding fundamental issues in cancer cell biology such as the role of environmental factors, both external factors such as UV exposure, air and water pollutants, viral infections, cigarette smoke, and internal factors such as how obesity and other metabolic disorders play a role in cancer development; how cancer cells grow; cancer gene–gene cooperation and cancer gene–environment interactions; factors that drive cancer progression from premalignant states through to metastasis; understanding mechanisms underlying cancer cell migration and invasion of neighboring normal tissues, in particular epithelial to mesenchymal transition (EMT); how cancers metastasize and target specific tissues; factors that determine how metastatic cancer cells can become dormant and resistant to chemotherapy and then awaken and cause cancer recurrence; the nature of cancer stem cell/progenitors in specific tissues; how cancer cells communicate with their neighboring stromal cells; how cancer cells develop chemoresistance; how cancer cells are influenced by and in turn influence local microbiomes; how the host immune system detects and responds to cancers, and how cancer cells adapt strategies to evade the host immune response, and how cancers can be detected at early stages that are more responsive to effective therapy.

The overall record of mouse models of cancer in addressing these questions is very good. There are many obvious strengths to mouse models, based on their decades-long use in cancer research and many successes and many mouse-based technologies in place. Thus, mice are clearly the predominant animal model for cancer research and will remain so for some time. Some of the advances in mouse-based technologies include: reverse genetic technologies such as homologous recombination (HR) in embryonic stem (ES) cells, patient-derived xenograft (PDX) models, and conditional and inducible gene expression systems; forward genetics technologies such as transgenes, bacterial artificial chromosomes (BACS), RNAi vectors, virus-mediated gene delivery, N-ethyl-N-nitrosourea (ENU) mutagenesis, transposons and transposon mutagenesis such as *Sleeping Beauty*, recombinant inbred (RI) and congenic mouse strains, humanized mouse models, mosaic mouse models, especially the development at the single cell level such as the mosaic analysis with double markers (MADM system) (GEMM mouse models are reviewed in [28]). In the MADM system, a gene can be mutagenized in a single cell along with an introduced marker to follow them in tumor development [29]. Importantly, the MADM system is very useful in recapitulating sporadic human tumors especially as it leaves intact interactions between a small number of tumor-initiating cells and surrounding normal stroma. A number of reporter systems for in vivo tumor imaging, e.g., luciferase, were first developed in mice. Finally, the genome, transcriptome, and proteome of mice are well characterized [30,31,32].

What are the limitations of mouse models? Overall, mouse models are more representative of human inherited genetic cancer syndromes, (e.g., familial adenomatous polyposis (FAP, *Apc* mutations), Lynch syndrome (mismatch repair mutations such as *MLH1*), breast cancer (*Brca1*, *Brca2*), familial endocrine and neural tumor syndromes, (e.g., neurofibromatosis 1 (NF1) than sporadic human cancers that account for the great majority (perhaps up to 90%) of human cancers and cancer deaths. Each of the various genetic mouse models brings different limitations [28,30]. For genetically engineered mouse models (GEMM) the host stroma does not recapitulate what occurs in humans whereby a small number of tumor cells interacts with a large number of normal stromal cells. For xenograft models in immunodeficient mice, there are differences in tumor histology with human tumors and the lack of a role for microbiota, plus they combine the background of mouse stroma and human tumor cells. Mouse tumors are far less heterogeneous than human tumors. While there have been a number of successes in using mouse models to develop anticancer therapies, (e.g., aspirin, NSAIDS) [31] many cancer drugs shown to be efficacious in mouse models have failed human clinical trials, e.g., endostatin [33], hedgehog inhibitors, MMP inhibitors, anti-CD28 Mabs (TGN1412), and cancer vaccines, or these drugs were found to be safe in mice but toxic in humans. Some of the reasons proposed for these limitations of mouse models include genetic, molecular, immunologic, and cellular differences between mice and humans, which makes sense given the vast species differences such as size, physiology, life span, and organ morphology [29,32]. An example is telomerase activity, as most adult mouse cells have active telomerase compared with adult human cells thus mouse cells tend to immortalize and transform more readily than do human cells, thus mouse cells require fewer genetic alterations for malignant transformation than human cells. Tumors in mice differ in several other ways from human tumors. Mouse tumors are not only less heterogeneous but metastases are rare in mice or they display different tissue specificity than human cancers suggesting that mechanisms underlying metastases might differ between the species. Mouse tumors tend to have different histology compared with human tumors of the same tissue type and mice with engineered mutations often develop different cancer spectrums, e.g., mice with constitutive heterozygous *Rb* mutations (*Rb* homozygous mutations are embryonic lethal in mice) only mainly develop pituitary adenosarcomas while children with inherited constitutive *Rb* mutations develop retinoblastomas [34]. Another example is *Trp53* constitutive KO mice that mainly develop sarcomas whereas human Li Fraumeni patients harboring *TP53* mutations primarily develop carcinomas [35]. It is noted that for both *Rb* and *Trp53*, the addition of additional conditional mutations was permissive for a broader cancer spectrum, closer to the human cancers caused by inherited mutations, however, these conditional mouse mutants do not resemble the normal development of cancers in human patients mutant for either *Rb* or *TP53*. Another example is that humans typically develop intestinal cancer in the colorectum (primarily arising from mutations in the *APC* gene and dysregulation of WNT/β-catenin signaling) while mice with similar pathway dysregulation, such as the *Apc^Min^* mouse, develop tumors primarily in the small intestine [36].

How do hamster models compare with and complement mouse models of cancer, currently and going forward? First of all, as was discussed in the introduction, there have been several unique chemically or virally induced hamster models that have been shown to be excellent models of human cancers. These include the oral cancer model with the unique large hamster cheek pouch that when treated topically with carcinogens such as DMBA develops oral cancers that advance through all stages of cancer development similar to the progression of human oral cancers [11,14,17,18,19,20]. There is also the chemically induced pancreatic cancer model that closely resembles human pancreatic cancer in the stages of cancer progression and in the genetic driver mutations [12,13,14,15,16]. Hamsters are also permissive to oncolytic adenovirus for testing gene therapies [13]. 

Certain human cytokines are functional in the hamster but not in the mouse, e.g., GM-CSF, IL-12, and MIF. An excellent example is the use of human interleukin 12 (IL-12), a potentially powerful agent for human anti-tumor immunotherapy [37]. Until recently, the risk of lethal toxicity has prevented IL-12 use in human cancer patients, thus there has been a need for an animal model to test new modified IL-12 therapies for safety and efficacy. Human IL-12 is non-functional in mouse models but highly functional in hamsters. In a recent study, human IL-12 was introduced into a hamster pancreatic cancer model using oncolytic adenoviruses that can replicate efficiently in hamsters and hamster cancer cell lines (again, unlike mouse models). Here, treatment of hamster pancreatic cancer was shown to be safe and effective as IL-12 therapy enhanced the survival of hamsters harboring orthotopic metastatic pancreatic cancer [37].

Regarding the various technological advances in mouse models of cancer, with the advent of CRISPR/cas9 genetic engineering in hamsters, many of the technologies available in mice are becoming available in hamsters [22,23,38]. These include conditional KOs and knockins using Cre recombinase activity, PDX and humanized hamster models, and hamster reporter strains such as a hamster luciferase line for imaging of hamster cancers. Finally, as we discuss below, genetically engineered hamster cancer models have already revealed unique cancer phenotypes that are closer to cancers that develop in humans and distinct from mouse models. It is early days yet in the development of genetically engineered hamster human diseases models, including cancer models, but there is already strong evidence that hamsters can complement or surpass mouse models for some human cancers or cancer sub-types.

We also note that there are no special nutritional or behavioral aspects to hamster husbandry that would impact the use of the hamster in cancer or other human disease studies. The nutrition needs of Syrian hamsters have been well studied and standard hamster diets have been developed from a close to a century-long history of using the hamster as a laboratory organism. To our knowledge, most commercial hamster distributors and laboratories use standard rodent diets to feed hamsters. We use the Laboratory Rodent Diet 5001 from LabDiet (St. Louis, MO, USA), a diet also developed for rats and mice, to feed our hamsters. The hamster has no special behavioral phenotypes which impact cancer phenotypes. Indeed, they are more gregarious in general than mice and easy to handle and when they develop cancer-related distress this is clearly discernable by their behavior in the cage. Hamsters are also provided with several enrichments in their environment such as large PVC tubes and special bedding.

What are the current limitations on hamsters as cancer models? With the hamster genome sequenced, albeit less annotated than the mouse genome, and genetic engineering techniques established, the major limitation in using hamsters is the relatively less availability of hamster reagents, including antibodies. However, motivated by the increasing use of hamsters in recent years, many laboratories and commercial entities have been actively developing hamster reagents. We expect that the availability of reagents for hamsters will be greatly improved. Nevertheless, the major consideration in choosing the hamster over other rodent species is for its better suitability of this organism for the biological questions to be addressed. For example, both humans and hamsters express CETP (cholesteryl ester transfer protein), a key gene in regulating reverse cholesterol transport (RCT), but neither mice nor rats carry this gene, making the hamster a better choice to study RCT.

We can also add that despite the recent major advances in hamster genetic engineering technologies still lag far behind mice in the breadth of hamster lines available to researchers, including the number of constitutive and conditional KO and knockin lines, including Cre recombinase transgene lines. Hamsters are not considered inbred, unlike virtually all of the background strains used to create mouse cancer models, although we note that isolated hamster colonies have become more genetically isogenic over time, and thus they are much less genetically diverse than wild hamsters. Therefore, the hamsters commonly used in laboratories, while not inbred, are genetically closely related. Inbred strains of hamsters have also been produced and used for scientific research but are not as readily commercially available as the outbred animals. Our laboratories have not tried to establish inbred wild-type hamster colonies, but among the over 20 genetically engineered hamster lines that we have produced, some of them have been bred over 8 years or over 10 generations; these lines should be even less genetically diversified. The lack of truly inbred hamster lines restricts the use of hamsters in many genetic studies.

Hamsters are also susceptible to the development of hamster polyoma virus (HaPV) which can cause lymphomagenesis. HaPV is endemic in hamster colonies worldwide and its presence can compromise cancer studies [39]. It is therefore essential for investigators to routinely test for the presence of HaPV in their colonies and to clean up colonies to eliminate HaPV when necessary (we note that hamster colonies in our laboratories have been maintained HaPV-free). 

Finally, hamster husbandry is more costly in all animal services facilities. The added costs in using hamsters over mice are largely due to two factors, lightly larger footprints of hamster cages and higher amounts of feed a hamster consumes. However, hamsters tend to have much larger litter sizes (10–12 pups/litter) than mice (4–8 pups/litter) and shorter gestation period (16 days) than mice (19–21 days), which makes hamsters easier and cheaper to produce, the costs of hamsters can be significantly offset.

## 3. Genetic Engineering in the Golden Syrian Hamster

Despite the long history of the golden Syrian hamster being used as a laboratory organism for biomedical research, the application of hamsters had been severely limited by the lack of technologies to manipulate the hamster genome. A few years ago, we took up the challenge to establish genetic engineering technologies in the hamster and successfully applied the CRISPR/Cas9 technique to conducting gene KO experiments in this species [22,23]. We subsequently also established CRISPR/cas9-mediated gene-knockin (KI) and edited techniques in the hamster. Recently, we also developed piggyBac-mediated transgenic techniques in the hamster and produced transgenic hamsters expressing human genes, including the human angiotensin-converting enzyme 2 (hACE2) transgenic hamster lines as a model for COVID-19 [40,41,42]. With these genetic engineering techniques, we have produced a long list of the first genetically engineered hamster lines which are being used by many laboratories in several countries to study a variety of aspects of basic biology and human conditions, including cancer, metabolic syndrome, circadian rhythm, and viral infections, which are reported in a companion review by some of us in this Special Issue (see the article by Liu and Wang in this special issue).

## 4. *KCNQ1*, *TP53*, and *IL2RG* Genetically Engineered Hamster Cancer Models

### 4.1. KCNQ1 Knockout Hamster Model

*KCNQ1* is a developmentally imprinted potassium channel gene that is widely expressed in both human and rodent tissues. Among other functions, KCNQ1 is well known for its voltage-gated interaction with its heterodimeric partner KCNE1 to regulate cardiac myocyte repolarization. *KCNQ1* mutations in humans cause a range of disease pathologies in humans including cardiac arrhythmia, inner ear defects, and gastric hyperplasia. Notably, work by our group has shown that *KCNQ1* acts as a tumor suppressor gene in the gastrointestinal tract in both humans and mice [43,44]. For example, the maintenance of the expression of KCNQ1 was associated with a significant survival advantage in human colorectal cancer patients with stage IV metastatic disease [44], and very recently it was reported that KCNQ1 was a major CRC prognostic predictor classifier as CRC patients with stage II and stage III CRC who maintained expression of KCNQ1 showed a much stronger disease-free survival (DFS) [45].

**Figure 1:**
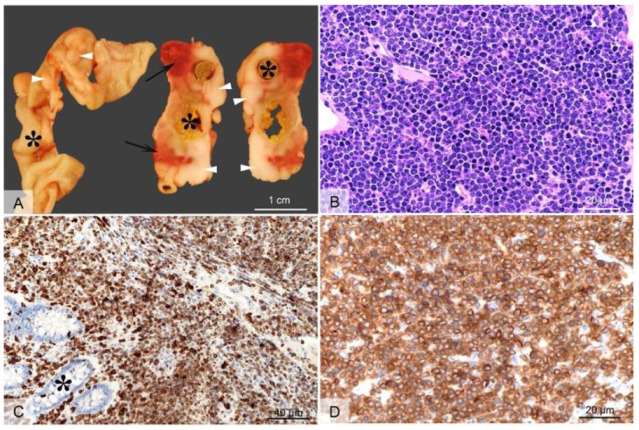
Gross and microscopic images of an intestinal T cell lymphoma in a *KCNQ1* homozygous mutant hamster. Grossly (**A**), extending from the intestinal wall through the serosa and mesentery, there are multifocal to coalescing white-beige masses (arrow heads), with focal areas of necrosis and hemorrhage (arrows). The tumors circumferentially surround the intestines (indicated by asterisks) which are occasionally moderately dilated and segmentally ulcerated (transmural). Histologically, the tumor consists of densely packed sheets of round to oval cells with scant basophilic cytoplasm (**B**) that infiltrate the intestinal mucosa ((**C**), asterisk) and subjacent layers through to intestinal serosa. The cells show a uniform, intense immunolabeling of plasma membrane for CD3 (**C**,**D**) (anti-CD3 antibody, DAKO/Agilent, Santa Clara, CA, USA; Cat# A0452). H&E, 40× objective Panel (**B**); CD3 immunolabeling with 20× objective Panel (**C**), and with 40× objective Panel (**D**) (from [46], with permission).

Employing CRISPR/cas9 gene targeting an 11-bp insertion in the hamster *KCNQ1* gene was introduced into the hamster genome, which resulted in a constitutive null allele [46]. *KCNQ1* homozygous KO hamsters demonstrated similar neurological defects and other phenotypes that are similar to *Kcnq1* KO mice [44], including inner ear defects, head bobbing and smaller stature [46]. Adult *KCNQ1* homozygous KO hamsters developed severe physical stress as early as 70 days of age, including overt cancers at necropsy [46]. Overall, >85% of the *KCNQ1* homozygous KO hamsters developed cancers, with the mean age when they became moribund at 150 days. None of the hamster littermate siblings that were either wild-type or heterozygous for *KCNQ1* mutations developed cancers. The four most common cancers observed following pathological analysis were T-cell lymphomas (the most common cancer), plasma cell tumors, hemangiosarcomas, and myeloproliferative disease consistent with myeloid leukemia [46]. Figure 1 depicts gross and microscopic images of an intestinal T-cell lymphoma.

The cancer phenotype in *KCNQ1* mutant hamsters was unique in several aspects. First, the *KCNQ1* KO hamsters represented the initial creation of a genetically engineered hamster cancer model. Second, while *Kcnq1* mutant mice demonstrated an enhanced intestinal cancer phenotype when introgressed into the *Apc^Min^* model of intestinal cancer [44], *Kcnq1* mutations alone in mice did not generate cancers in any tissue, unlike hamsters, who developed a range of cancers, sometimes multiple synchronous cancers, in different tissues. Similarly, humans who are mutant for *KCNQ1* do not develop cancer, aside from the development of gastric hyperplasia which can represent a premalignant stage of gastric cancer. Thus, for reasons that remain unknown, the complete loss of KCNQ1 function uniquely leads to widespread blood cell cancer development in hamsters. How these cancers arise in *KCNQ1* KO hamsters is an area of active investigation. Studies in humans and mice have shown that KCNQ1 is expressed in several types of hematopoietic cells, including thymic T cells, bone marrow, and other white blood cells, although KCNQ1 expression patterns in these cell types in hamsters have not been adequately characterized. Work by our group and others has indicated that KCNQ1 may be involved in the regulation of tissue stem cell transformation. This has been proposed in GI cancers associated with bidirectional Wnt/β-catenin signaling. Work by our group has shown that *Kcnq1* mRNA [44] and protein expression (P. Scott et al., unpublished) was localized to the intestinal stem cell compartment. Further, *Kcnq1* expression has been reported in a murine bone marrow-derived stem cell progenitor population. Ongoing studies are exploring whether Wnt/β-catenin signaling is dysregulated in *KCNQ1*-deficient bone marrow-derived hamster progenitor cells, of particular importance with the emerging evidence of a significant role for Wnt signaling in hematopoietic cancers.

### 4.2. TP53 Knockout Hamster Model

**Figure 2:**
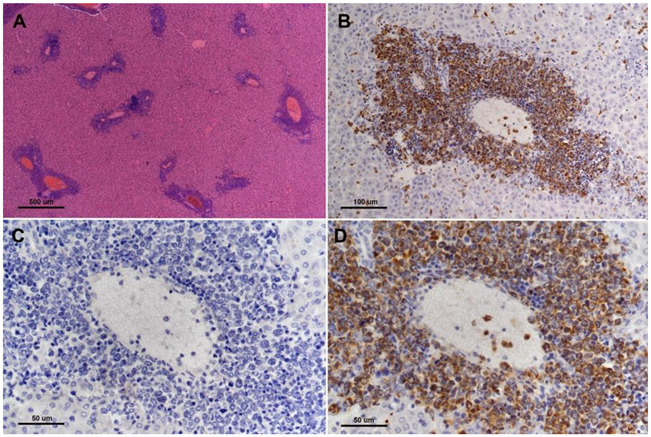
Myeloid leukemia involvement of liver of TP53 homozygous mutant hamster. There are multifocal infiltrates of neoplastic cells predominantly involving the portal triads ((**A**), H&E, 4× objective lens). Infiltrating cells surrounding hepatic portal veins are strongly myeloperoxidase positive ((**B**,**D**), 20× and 40× objective lens, respectively). (**C**) shows corresponding negative control (MPO-specific primary antibody is substituted with normal rabbit immunoglobulin on a serial step section, 40× objective lens) for image in (**D**) (from [49], with permission).

*TP53* is considered the canonical tumor suppressor gene, commonly mutated in most major human cancers, and for this reason, it has been referred to as the “guardian of the genome” [47]. TP53 is a sequence-specific transcription factor that is involved in a range of cellular functions that are critical for its role in suppressing cancer, including cell cycle regulation, sensing of DNA damage, sensing and reaction to oxidative stress, apoptosis, senescence, and cellular metabolism [48]. Employing CRISPR/cas9 genetic engineering we created a 1-bp insertion in the hamster *TP53* gene at amino acid position 311 that resulted in a truncation mutation that disrupts the DNA binding domain of TP53 [49]. We found that both *TP53* homozygous and *TP53* heterozygous KO hamsters developed a wide range of cancers starting at 53 days of age, with individual hamsters often manifesting multiple synchronous cancers in different tissues. In contrast, a control group of *TP53* wild-type siblings did not develop cancer, even when aged beyond one year. On average, *TP53* homozygous mutants survived 139 days, and *TP53* heterozygous mutants survived 286 days. We analyzed by histopathology 52 *TP53* homozygous mutant hamsters and 30 *TP53* heterozygous mutant hamsters [49]. The homozygous mutant hamsters developed a wide range of cancers, with lymphomas (29%), hemangiosarcomas (27%), and myeloid leukemias (21%) representing the most common cancers. To a lesser extent, they developed several other sarcomas and adenocarcinomas in the adrenal glands, pancreas, and kidney. In *TP53* heterozygous hamsters, lymphomas (67%) were the predominant type of cancer, followed by hemangiosarcomas (17%), myeloid leukemias (6%), and several other sarcomas and carcinomas that were found in only one hamster. Notably, in a group of 17 cancers from *TP53* heterozygous animals all showed loss of heterozygosity (LOH) at the *TP53* locus [49]. See Figure 2 depicts myeloid leukemia involvement in the liver of *TP53* mutant hamsters.

The cancer phenotypes in TP53 mutant hamsters were notable in several areas. First of all, in contrast with *Trp53* homozygous KO mice, hamsters developed carcinomas in several epithelial tissues that were not observed in *Trp53* KO mice. Human Li Fraumeni patients who carry germline *TP53* mutations typically develop carcinomas while *TP53* null mice primarily develop sarcomas. *Trp53* mice carrying gain of function point mutations do develop a broader range of cancers that is closer to human cancers with *TP53* mutations and *TP53*-deficient hamsters with one major exception in that they do not develop myeloid leukemias, which are common in *TP53* mutants (especially homozygous mutant) hamsters and in human myeloid leukemia patients. *TP53*-deficient acute myelogenous leukemia (AML) is a particularly aggressive sub-type of AML with a median survival of 5–9 months and one-year survival of 0–10% [50]. Further, *TP53* mutations are common in therapy-related AML (t-AML), a very aggressive disease because of the high likelihood of treatment failure. *TP53* mutations are also found in 20–27% of AML with myelodysplasia-related changes (AML-MRC) and up to 40% of therapy-related AML myelodysplastic syndrome (AML/MDS) [50]. *TP53* mutations are also associated with high-risk MDS and rapid transformation to secondary AML (sAML), an extremely lethal disease with less than 6 months median overall survival [50]. There are currently few treatment options for *TP53*-deficient AML and one of the major barriers to the development of new therapies has been the lack of a reliable rodent model. *Trp53*-deficient mice do not develop AML, but now there is a hamster model that can be used to study the genetic steps that cause this disease, including high-risk MDS that can rapidly transform to secondary AML. Hamsters can now also be used to test new drug modalities to treat *TP53*-deficient AML.

### 4.3. IL2RG KO Hamster Model

*IL2RG* encodes the commonly shared gamma chain subunit in the interleukin-2 receptors (IL2Rs) which meditate the functions of multiple type I interleukin cytokines, including interleukin-2 (IL-2), IL-4, IL-7, IL-9, IL-15 and IL-21 [51,52]. *IL2RG* resides on the X-chromosome and loss of function mutations in this gene cause X-linked severe combined immunodeficiency (XSCID) which is characterized by a failure in T development, lack of class switching recombination of immunoglobin genes in B lymphocytes, and the absence of natural killer (NK) cells. These severe immunodeficiency features highlight the importance of IL2RG in the development of adaptative immunity and innate immunity.

To develop a hamster model for XSCID, as well as a host for human tumor engraftment (discussed below), we recently created several *IL2RG* KO hamster lines. To genetically inactivate *IL2RG* in the hamster, we employed the CRISPR/cas9 system with single guide RNAs (sgRNAs) designed to target its exon 1. Among the produced hamster lines carrying different insertions and deletions (indels) in exon 1, lines with frameshift mutations causing multiple premature stop codons in *IL2RG* were chosen for the establishment of *IL2RG* KO hamster colonies and used to produce experimental animals [38,53]. Lymphocyte-specific gene expression analyses in the spleen showed that *IL2RG* KO hamsters present severe defects in lymphocyte development which are characterized by a greatly reduced number of CD4^+^ T cells and barely detectable CD8^+^ T cells, B cells, and NK cells, mimicking many aspects of human XSCID immunodeficiency. The use of these *IL2RG* KO hamsters as an XSCID model to study the infectious diseases that are of great health concern in immunocompromised patients was reported by us [52]. Here, we summarize our recent studies in using the *IL2RG* KO hamsters as a host for human tumor tissue engraftment to produce patient-derived xenograft (PDX) models as laboratory avatars for oncology research.

The use of immunodeficient animals, either with IL2RG-deficiency alone or in combination with other immunodeficiencies such as RAG2-deficiency, as hosts to produce PDX models relies on their incapability of mounting immune rejections to human tissues, therefore allowing them to be xenografted in the animal hosts. Compared to in vitro cultured tumor-derived cell lines where in vitro adaptation takes place which may change the physiology of tumors, the PDX models better recapitulate the tumor biology in vivo [53]. *Il2rg* KO mice have been widely used as hosts for producing PDX models [54]. However, there are several significant limitations in using mice as hosts for human tissue xenografting, chief among which are the incompatibility of mouse growth factors and cytokines with human tissues and cells, leading to low xenografting rates, limited or no self-renewal of malignant stem cells, and low cancer cell metastasis rates. The lack of functional communications between human cells/tissues and the mouse physiological milieu is also one of the underlying causes of the fundamental problems in using immunodeficient mice as hosts for human hematopoietic stem cell transplantation to produce so-called humanized mice carrying a humanized hematolymphoid system. Due to the lack of functional support from mouse cytokines for human lymphoid and myeloid cells, mice are incapable of fully supporting the development of a human immune system, as manifested by impaired lymph node development, poorly developed germinal centers, defective humoral immune responses (characterized by low levels of immunoglobulin production and inefficient immunoglobulin class switching recombination) and human NK cell development [55,56,57,58,59]. To address this species incompatibility issue, next-generation humanized mice transgenically expressing human cytokine genes, such as *IL-3* and granulocyte macrophage-colony stimulating factor (*GM-CSF*) that are critical for myeloid cell development [60], and other human genes such as in the MISTRG mice [61], have been developed. The transgenic expression of these human genes has significantly improved the engraftment of human tumor tissues for mouse PDX model production [62] and the development of human lymphoid and myeloid cells. Nevertheless, these humanized mice are still not optimal in several aspects including being defective in mounting antigen-specific adaptive immune responses, low levels of reconstitution of gut-associated lymphoid tissues from human cells, and poor lymphoid architecture and organ development [57].

Therefore, a small rodent with better physiological compatibility with human cells/tissues is highly desired as a host for PDX and immune system humanization. In this regard, the golden Syrian hamster is a promising candidate, as in contrast to mice several human cytokines such as GM-CSF [63] and IL-12 [37,64] are cross-reactive with hamster cells. As mentioned above, human GM-CSF is among the key cytokines transgenically expressed in the MISTRG mice, which greatly improved human myeloid cell reconstitution [60]. To test whether the hamster is indeed a suitable host for human PDX, we carried out human pancreatic cancer cell transplantation experiments in the *IL2RG* KO hamsters and produced the first hamster PDX model. In these experiments, we used an *IL2RG* KO line (named ZZU001) that we generated in which a 10 bp frameshift deletion in exon 1 fully inactivated the expression of the *IL2RG* gene [38].

**Figure 3:**
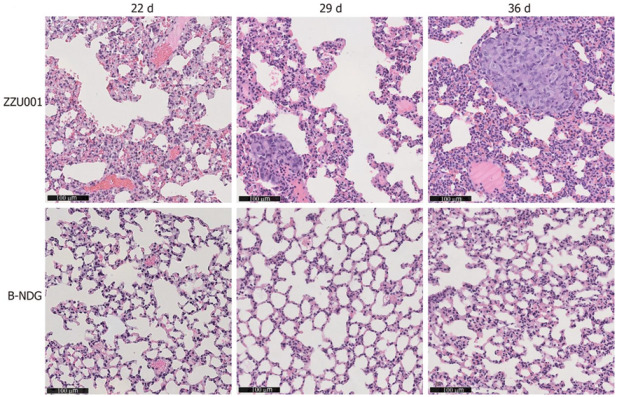
(From [38], with permission). H&E staining of a lung metastasis at 22 d, 29 d, 36 d after subcutaneous injection of MIA-PaCa-2 cells in ZZU001 hamsters and B-NDG mice. Scale bars = 100 µm.

We first performed subcutaneous xenotransplantation of a human pancreatic cancer cell line, MIA-PaCa-2 [65], into ZZU001 hamsters, and for comparisons, also into immunodeficient B-NDG (NOD-*Prkdc^scid^ IL2rg^tm1^*/Bcgen) mice which are defective in both the *IL2rg* gene and the *Prkdc* gene. While xenografted human pancreatic cancer cells developed in both the hamster and B-NDG mouse hosts, no distant metastasis was identified in mice but was found in the lung, kidneys, and adrenal glands in ZZU001 hamsters (Table 1). Figure 3 (From [38]) shows a H&E staining of a lung metastasis at 22 d, 29 d, and 36 d after subcutaneous injection of MIA-PaCa-2 cells into ZZU001 hamsters and B-NDG mice. The same observations, i.e., metastasis was only observed in the hamster hosts but not in the mouse hosts, were also made from each of the other four independent human pancreatic cancer cell lines tested (Table 2).

Because orthotopic transplantation models mimic the biology of primary tumors more closely than subcutaneous transplantation models, to establish the feasibility of the hamster as an orthotopic model for human pancreatic cancer cell transplantation, orthotopic transplantation experiments with MIA-PaCa-2 cells were also performed in both ZZU001 hamsters and B-NDG mice. MIA-PaCa-2 cells were directly injected into the pancreas of hamsters and mice and tumor formation rates, as well as tumor metastasis, were compared between these two host species. Orthotopic transplantation of Mia-PaCa2 cells in hamsters led to a 100% (5/5) tumor formation rate and metastasis at multiple sites, including liver (100%), lung (100%), retroperitoneum (100%), mesentery (100%), kidney (100%), diaphragm (40%), adrenal gland (40%), and stomach (20%). In contrast, much lower metastatic rates to other organs were observed, with no lung metastasis observed at all, in the B-NDG hosts (Table 1). In addition, metastatic tumors developed in the hamster orthotopic models presented similar clinical and pathological features to what was observed in human patients, which includes local infiltration of cancer cells, ascites, jaundice, ileus, and cachexia (Table 1).

In summary, we demonstrated that in comparison to the highly immunodeficient B-NDG mice, ZZU001 hamsters with the loss of *IL2RG* function alone (therefore much less immunodeficient than B-NDG mice) support better human pancreatic cancer cell engraftment and multiple organ metastasis and serve as an improved host for human pancreatic cancer engraftment. We posit that the improved engraftments and higher metastasis rates in the ZZU001 hamsters than in mouse hosts by human cancer cells are attributed to better communications between the human cancer cells with the hamster physiological milieu.

## 5. Summary

There is no perfect rodent cancer model. Early cancer studies that go back as far as the 1930s employed a range of small test animals, such as rabbits, rats, hamsters, gerbils, and mice, primarily based on the use of chemical carcinogens. However, since the 1980s mice largely supplanted other rodents in cancer research due to several factors: the development of inbred strains for genetic studies; lower costs; but perhaps most importantly, due to their amenability to genetic engineering, such as via the introduction of oncogenic transgenes and the creation of KO and knockin (KI) models following genetic manipulation in embryonic stem (ES) cells, a characteristic that until recently was unique to mouse models. One can also add recent advances in mouse model technology including PDX and humanized models. Overall, mouse models of cancer have proven invaluable in understanding carcinogenesis at the genetic, biochemical, molecular, and pathological levels. However, as discussed above, mouse models also present significant limitations in understanding human cancers and the development of therapeutic modalities to treat these cancers. Part of the reason for these limitations is due to species differences in size, lifespan, physiology, organ morphology, and other known and unknown factors such as the natural resistance of mice to the spontaneous development of cancers and resistance to the development of the most aggressive forms of cancer. In this review, we propose that the application of new genetic engineering technologies based on CRISPR/cas9 gene targeting in other rodent models, here, the golden Syrian hamster, provides an opportunity to expand the suite of rodent cancer models that can complement and improve upon work being performed in mouse models. See Table 3, which provides a list of the various hamster cancer models discussed in this review. Hamsters have long been used as superior rodent models for various human disorders including infectious disease (including currently SARS-CoV-2), dyslipidemias, and diet-induced obesity but also several chemically induced cancer models such as the DMBA-induced oral cancer model. Now, the first generation of CRISPR/Cas9 gene-targeted hamster disease models have been created including the first three KO cancer models, *TP53*, *KCNQ1*, and *IL2RG*. These models have already revealed unique cancer phenotypes in hamsters that can better inform on human cancers. An example is the development of aggressive *TP53*-deficient AML, a cancer with an extremely poor prognosis in humans that is largely resistant to current therapy. A total of ~30% of *TP53* KO hamsters develop AML, in contrast with *Trp53* KO mice that are fully resistant to the development of AML. Similarly, *KCNQ1* KO hamsters developed a wide range of lethal cancers while *Kcnq1* KO mice fail to develop cancers at all. Finally, we have demonstrated that *IL2RG* KO hamsters can be a superior rodent model for metastatic human PDX studies.

In summary, understanding the genetic, biochemical and other factors underlying species differences in cancer susceptibility and response to therapy between multiple rodent models and humans can significantly complement and enhance the utility of rodent models in cancer research leading to advances in areas such as cancer gene discovery, biomarker discovery, and cancer therapeutics.

## Figures and Tables

**Table 1:** (From [38], with permission). Metastasized organs and metastasis frequency by MIA-PaCa-2 cells transplanted subcutaneously (SC) and orthotopically (OrT) in hamsters and mice.

	MIAPaCa-2 (SC)		MIAPaCa-2 (OrT)	
	B-NDG	ZZU001	B-NDG	ZZU001
Distant metastasis				
Liver	-	-	3/5 (60)	5/5 (100)
Lung	-	5/5 (100)	-	5/5 (100)
Retroperitoneum	-	-	3/5 (60)	5/5 (100)
Mesentery	-	-	3/5 (60)	5/5 (100)
Diaphragm	-	-	2/5 (40)	2/5 (40)
Spleen	-	-	2/5 (40)	-
Stomach	-	-	-	1/5 (20)
Kidney	-	2/5 (40)	-	5/5 (100)
Adrenal gland	-	1/5 (20)	-	2/5 (40)
Local infiltration				
Spleen	-	-	3/5(60)	4/5 (80)
Stomach	-	-	1/5 (20)	3/5 (60)
Liver (hilus)	-	-	3/5 (60)	5/5 (100)
Kidney (hilus)	-	-	-	1/5 (20)
Retroperitoneum	-	-	3/5 (60)	5/5 (100)
Bowel	-	-	3/5 (60)	5/5 (100)
Mesentery (adjacent to pancreas)	-		4/5 (80)	5/5 (100)
Signs of tumor burden				
Ascites	-	-	3/5 (60)	3/5 (60)
Jaundice	-	-	2/5 (40)	3/5 (60)
Ileus	-	-	-	2/5 (40)
Cachexia	-	-	1/5 (20)	3/5 (60)

**Table 2:** (From [38], with permission). Metastasized organs and metastasis frequency by four independent human pancreatic cancer cell lines transplanted subcutaneously (SC) in hamsters and mice.

	Panc-1 (SC)	SUIT-2 (SC)	Patu8988T (SC)	Capan-1 (SC)
	ZZU001	ZZU001	ZZU001	ZZU001
Distant metastasis				
Liver	-	1/3 (33)	3/5 (60)	-
Lung	5/5 (100)	3/3 (100)	-	5/5 (100)
Retroperitoneum	-	-	-	-
Mesentery	-	-	-	-
Diaphragm	-	-	-	-
Spleen	-	-	-	-
Stomach	-	-	-	-
Kidney	-	1/3 (33)	1/5 (20)	-
Adrenal gland	-	-	-	-

**Table 3:** Representative literature on hamster cancer models discussed in this review.

Head and Neck Cancer	2008 Review of the Hamster Model of Sequential Oral Oncogenesis [11]
	Results of the effect of smokeless tobacco on oral microbiota in the hamster cheek pouch carcinogenesis model [15]
	Salivary exosome proteomics and bioinformatics analysis of DMBA-induced oral cancer with radiation therapy in the hamster oral carcinogenesis model [17]
	Hamster cheek pouch model of oral cancer for boron neutron capture therapy studies: Selective delivery of boron by boronphenylalanine [18]
	A 2020 study describing optimization of the oral cancerization model in hamsters to study oral cancer therapy [19]
	A 2019 review of the hamster model of sequential oral carcinogenesis [20]
Pancreatic cancer	A 2011 review of the use of hamsters for chemically induced pancreatic cancer, use in prevention, treatment and relevance to the human disease [12]
	Effect of use of Fucoxanthinol on BOP-treated pancreatic ductal adenocarcinoma cells in a hamster pancreatic cancer model [16]
	IL2RG knockout created by CRISPR/cas9 technology for creation of PDX metastatic pancreatic cancer models—described in detail in this review [38]
Other chemicallyinduced cancers	Very early (1961) study employing 20-Methylcholanthrene oral administration induction of intestinal, mammary and ovarian cancers [10]
Oncolytic adenoviruses	Chapter review by Wold and Toth, pioneers in this field, from 2012 that summarizes the use of the hamster as an animal model to study oncolytic adenoviruses and to evaluate the efficiacy of antiviral compounds [13]
	Describes the use of an oncolytic adenoviral vector to express IL-12 to treat chemically induced pancreatic cancer in the hamster [64]
Hamster polyoma virus	A very recent review of HaPV research, including the prevalence of HaPV in hamster colonies worldwide and the risk of lymphomas in HaPV positive hamsters [39]
*KCNQ1* Knockout	Knockout generated by CRISPR/cas9 technology, develop a wide range of cancers, with the top four cancers being T-cell lymphomas, plasma cell tumors, hemangiosarcomas and myeloproliferative disorders, discussed in detail in this review [46]
*TP53* Knockout	Knockout generated by CRISPR/cas9 technology, develop a wide range of cancers, with the top three cancers being lymphomas, AML and hemangiosarcomas, discussed in detail in this review [49]

## Data Availability

Not applicable.
